# High and low dose radiation effects on mammary adenocarcinoma cells – an epigenetic connection

**DOI:** 10.18632/oncoscience.298

**Published:** 2016-03-10

**Authors:** Lidia Luzhna, Jody Filkowski, Olga Kovalchuk

**Affiliations:** ^1^ Department of Biological Sciences, University of Lethbridge, Lethbridge, AB, Canada

**Keywords:** apoptosis, quantitative ultrasound, cancer therapy, treatment response monitoring, personalized medicine

## Abstract

The successful treatment of cancer, including breast cancer, depends largely on radiation therapy and proper diagnostics. The effect of ionizing radiation on cells and tissues depends on the radiation dose and energy level, but there is insufficient evidence concerning how tumor cells respond to the low and high doses of radiation that are often used in medical diagnostic and treatment modalities. The purpose of this study was to investigate radiation-induced gene expression changes in the MCF-7 breast adenocarcinoma cell line. Using microarray technology tools, we were able to screen the differential gene expressions profiles between various radiation doses applied to MCF-7 cells. Here, we report the substantial alteration in the expression level of genes after high-dose treatment. In contrast, no dramatic gene expression alterations were noticed after the application of low and medium doses of radiation. In response to a high radiation dose, MCF-7 cells exhibited down-regulation of biological pathways such as cell cycle, DNA replication, and DNA repair and activation of the p53 pathway. Similar dose-dependent responses were seen on the epigenetic level, which was tested by a microRNA expression analysis. MicroRNA analysis showed dose-dependent radiation-induced microRNA expression alterations that were associated with cell cycle arrest and cell death. An increased rate of apoptosis was determined by an Annexin V assay. The results of this study showed that high doses of radiation affect gene expression genetically and epigenetically, leading to alterations in cell cycle, DNA replication, and apoptosis.

## INTRODUCTION

Ionizing radiation kills cells by damaging their DNA. Radiation induces a variety of DNA lesions, such as damage to nucleotide bases, cross-linking, and DNA single- and double-strand breaks [1]. Radiation can damage normal cells, as well as cancer cells, and is often used in medical diagnostic and treatment procedures. Any use of ionizing radiation, therefore, must be carefully planned to minimize side effects and deliver optimal results. Diagnostic imaging procedures use low doses of radiation, whereas radiation therapy uses high energy radiation to shrink tumors. About half of all cancer patients receive radiotherapy during the course of their treatment, and all cancer patients are exposed to diagnostic-related radiation. Although the benefits from the medical procedures greatly outweigh any potential low risk of harm, more evidence has been found to prove that harm from diagnostic X-rays is linked to an increased risk of cancer. This harm is correlated to the radiation dose absorbed[2].

A radiation dose is the amount of energy absorbed by the body in radiation interactions. Different types of radiation may produce different biological effects, and the magnitude of the effect varies according to the dose rate[2]. Stochastic effects of radiation, such as cancer and hereditary effects, are caused by mutations and other permanent changes in which a cell remains viable. The probability of such stochastic effects increases with dose (no threshold), but the severity of the outcome is not related to the dose[3]. Nevertheless, epidemiologic studies continue to reveal cancer risks associated with diagnostic radiologic procedures[2]. Oh and Koea provided an overview of radiation-related cancer risk associated with multiple computed tomographic (CT) scans that were required for follow up in colorectal patients. Of 36 studies analyzed in their review, 34 showed a positive association between medical imaging radiation and increased cancer risk, although the radiation risk from low doses was uncertain[4].

Significant dose-response relationships were found for breast cancer risk for patients with tuberculosis who received frequent fluoroscopy[5, 6]. Furthermore, there is a statistical association between radiation doses and types of diagnostic X-ray examinations and chromosome translocation frequency[7, 8], whereby high doses of radiation are more successful in killing cells, and low doses contribute to mutational events that lead to carcinogenesis.

Moreover, the vast majority of low-dose radiation effects and radiation-induced cancer studies have been conducted on non-cancerous tissues. Very little is known about the effects of low-dose diagnostic radiation exposure in actual cancer cells and tissues. It is possible that low doses of radiation can contribute to the genomic instability of cancer cells, leading to an increase in malignancy and potentially making cancer cells resistant to further radiation with higher doses.

One of the major obstacles to successful cancer management is acquired resistance to radiation therapy. The mechanisms of such resistance have considerable clinical significance but are poorly defined. The limitation of radiotherapy is that solid tumor cells often become deficient in oxygen after radiation exposure. Such tumors can outgrow their blood supply, causing hypoxia[9]. Under hypoxic conditions, cancer cells can become two to three times more resistant to radiation. There are also several extra-nuclear factors that cause resistance to radiation. The levels of IGF-IR and its substrate are elevated in ER- positive breast tumors and can be linked with increased radio-resistance and cancer relapse[10].

MCF-7 breast carcinoma cells are known to be resistant to radiation-induced apoptosis due to the lack of caspase-3, and apoptosis is independent of cell cycle control[11]. Radioresistance is also common in chemoresistant cancer cells. For example, MCF-7/Pac and MCF-7/Doc were found to be radioresistant to γ-radiation, and MCF-7/DOX cells showed increased resistance to X-rays[12, 13]. According to Zhang and colleagues, lower doses of ionizing radiation led to inhibition of HIF-1 (the transcription factor involved in the process of hypoxic adaptation of neoplasm), whereas higher doses increased HIF-1α, HPSE-1, EEGF, and CD31 levels in irradiated mice[14]. Because the response to ionizing radiation correlates with the existence of oxygen that forms DNA-damaging free radicals, hypoxic regions in tumors require higher radiation doses to obtain the same damage as normoxic regions. Certain factors, including HIF-1α, improve tumor adaptation to hypoxia and are involved in radioresistance[14].

Based on the information in the literature, we concluded that the effect of ionizing radiation on cells and tissue is dependent on the radiation dose and energy level, but there is not enough evidence on how the tumor cells respond to low and high doses of radiation, which are often used in medical diagnostic and treatment modalities. Therefore, the aim of this study was to investigate the response of MCF-7 breast carcinoma cells to low, medium, and high X-ray doses and to define any radiation-associated changes in gene expression and apoptosis levels.

## RESULTS

### Effect of low, medium, and high doses of radiation on whole genome gene expression in MCF-7 cells

**Figure 1:**
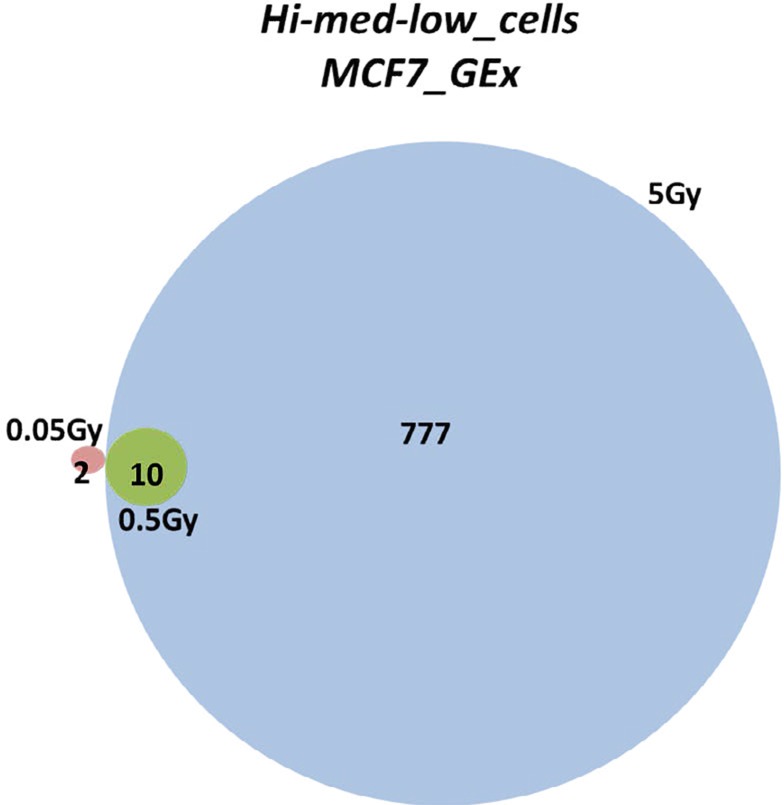
Gene expression profiling of MCF-7 breast adenocarcinoma cells The Venn diagram shows the number of significantly changed genes in the MCF-7 cell line with low (0.05 Gy), medium (0.5 Gy), and high (5 Gy) doses of radiation, in comparison to their corresponding non-irradiated controls, as identified by the gene expression profiling analysis

**Figure 2:**
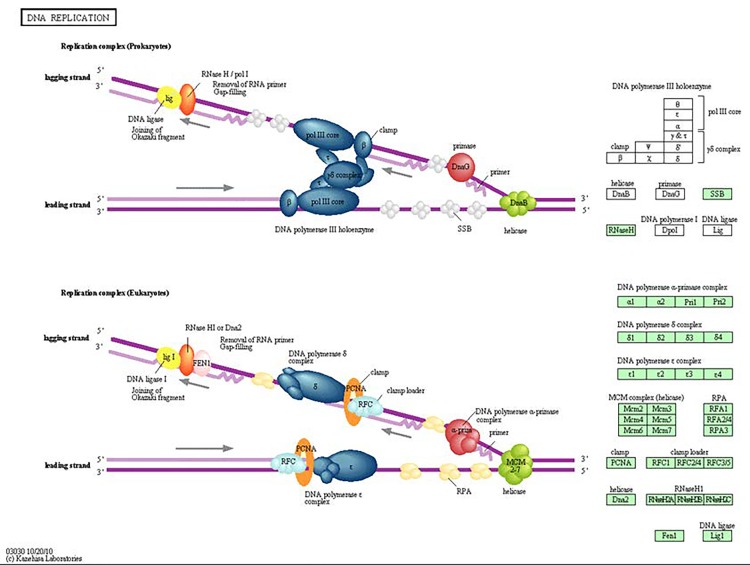
The KEGG DNA replication pathway All encircled genes were downregulated.

We isolated RNA from MCF-7 breast adenocarcinoma cells and performed gene expression profiling. A drastic difference in the radiation-induced gene expression changes was discovered between the doses applied. Only high doses of X-ray exposure led to dramatic alterations in gene expression, whereas low and medium doses did not affect gene expression. A total of 2, 10, and 777 genes were affected by the 0.05, 0.5, and 5 Gy of radiation, respectively (Figure 1). Further, we evaluated the 777 genes that changed their expression level and found: 437 genes were upregulated and 340 genes were downregulated. With the help of the DAVID functional annotation array analysis tools, we were able to identify and group the evaluated genes according to their function and possible role in certain pathways. Subsequently, genes with similar or identical functions were grouped together, and based on their expression changes, the role of certain pathways in radiation response was evaluated (Table 1). Twenty-nine cell cycle genes and twenty-one genes involved in DNA replication were downregulated (Table 1, Figure 2). The primary repair processes were shut down by the inhibition of the expression of key genes. MCF-7 cells may harbors altered activity of MMR, NER, BER, and HR due to the downregulation of the 9, 12, 8, and 6 pathway genes, respectively (Table 1). Such changes are often associated with cell death. Furthermore, the genes responsible for apoptosis from the p53 signaling pathway were upregulated (Table 1).

**Table 1:** The significantly altered KEGG pathways in MCF-7 cells after 5 Gyof X-ray treatment in comparison to the corresponding untreated controls In this table, the pathway significance (%) is defined as the ratio of gene alterations that similarly affect a certain pathway (either up- or downregulate) to the total number of altered genes in the pathway. “↑” – the pathway is upregulated; “↓” – the pathway is downregulated.

Pathways	# Genes changed	Pathway direction and confidence
DNA replication	21	↓95.2%
Cell cycle	29	↓96.6%
Nucleotide excision repair	12	↓66.7%
Mismatch repair	9	↓88.9%
p53 signaling pathway	12	↑83.3%
Glutathione metabolism	10	↑88.9%
Base excision repair	8	↓87.5%
Oocyte meiosis	13	↓84.6%
Homologous recombination	6	↓83.3%
Arginine and proline metabolism	8	↑87.5%
Pyrimidine metabolism	10	↓80.0%
Other glycan degradation	4	↑100%
Progesterone-mediated oocyte maturation	8	↓85.7%

**Figure 3:**
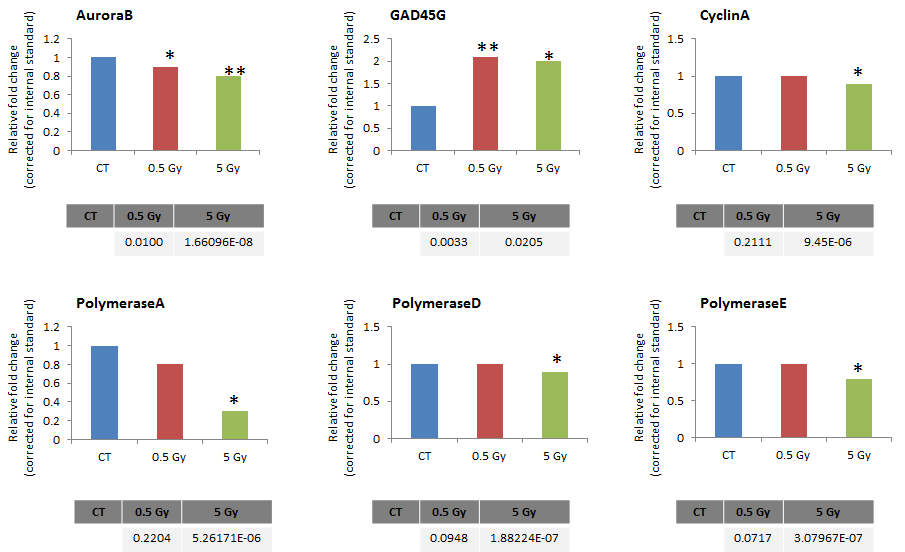
Altered levels of gene transcripts of Aurora B, Cyclin A, GADD45G,and polymerases A, D, E, as detected by RT-PCR Data are shown as fold changes to respective controls. Each treatment group was compared to its corresponding control; 18SrRNA was used as a reference gene (calculated by Pfaffl). *P*-values (in tables below the graphs) were calculated by Student's t-test.

We further performed the qRT-PCR analysis to confirm the validity of gene expression profiling on the following genes: DNA polymerases A, D, and E, Cyclin A (*CCNA*), *GADD45G*, and Aurora B (*AURKB*). The expression level of the *AURKB* gene in MCF-7 cells significantly decreased after application of 0.5 and 5 Gy of X-rays (Figure 3). Aurora B is a protein kinase that participates in a proper segregation of sister chromatids during the anaphase of mitosis. The *CCNA* transcript level decreased only with an application of the high radiation dose of 5 Gy (Figure 3). Cyclin A is necessary for the S phase of the cell cycle and its deficiency is often related to cell cycle arrest. Similarly, the expression levels of the DNA polymerases A, D, and E declined after 5 Gy of X-ray exposure (Figure 3). The *GADD45G* transcript level in MCF-7 cells was significantly increased after 0.5 and 5 Gy of X-rays exposure (Figure 3). It is known, that *GADD45G* (growth arrest and DNA-damage-inducible protein) is a marker of cell growth arrest and its level increases after treatment with DNA-damaging agents.

### miRNA expression in irradiated MCF-7 breast adenocarcinoma cells

In search of the possible regulators of gene expression, we proceeded to analyze the role of miRNAs in the radiation response of MCF-7 cells. miRNAs are involved in epigenetic control of gene expression regulation through the RNA interference pathway. miRNAs negatively affect the levels of their target transcripts and the levels of proteins encoded by these transcripts. In this way, miRNAs contribute to gene silencing, and changes in miRNA expression are common in cancers and in response to radiation.

We identified that one, three, and six miRNAs were significantly changed after exposure to 0.05, 0.5, and 5 Gy of X-rays, respectively (Table 2). miR-106a was significantly downregulated in a dose-dependent manner after all three radiation doses. Its putative target is the RB1 protein, which regulates cell cycle and promotes cell cycle arrest. Five Gy of radiation led to downregulation of miR-17 and miR-106b, which target BIM and p21 apoptosis inducing factors, whereas miR-23b and miR-149, targeting NOTCH (cell signaling pathway) and AKT (promotes proliferation), were upregulated (Table 2). Thus, changes in miRNAs expression seem to contribute to cell cycle arrest and initiation of apoptosis in MCF-7 cells exposed to ionizing radiation, influencing cellular stress response, and this response is dose dependent.

**Table 2:** Radiation-induced microRNA expression changes in MCF-7 cells Relative miR expression values are represented in folds in the irradiated cells in comparison to non-irradiated control cells, as analyzed by miRNA microarray. The significance of differences was analyzed by the Student's t-test.

MiRNA changed	Fold @ 0.05 Gy	Fold @ 0.5 Gy	Fold @ 5 Gy	Validated Target
23b			0.48	Notch
149			1.77	AKT
17			−0.85	BIM, p21, VEGF
106b			−0.72	P21, VEGF
106a	−0.25	−0.42	−0.93	VEGF, RB1
20a			−0.91	VEGF
let7a		1.14		Dicer
let7b		0.66		CDK6

### Radiation-induced apoptosis in MCF-7 breast adenocarcinoma cells

IR exposure is known to induce apoptotic cell death in irradiated cells. Therefore, we analyzed the levels of IR-induced apoptosis in MCF-7 cells. Early apoptosis is characterized by various changes in the cellular plasma membrane; the primary change is the translocation of phosphatidylserine (PS) from the inner layer to the surface of the membrane. Annexin V possesses a high affinity to PS, and this allows for the early detection of apoptotic changes[15]. Here, we analyzed IR-induced apoptosis using an Annexin V assay.

**Figure 4:**
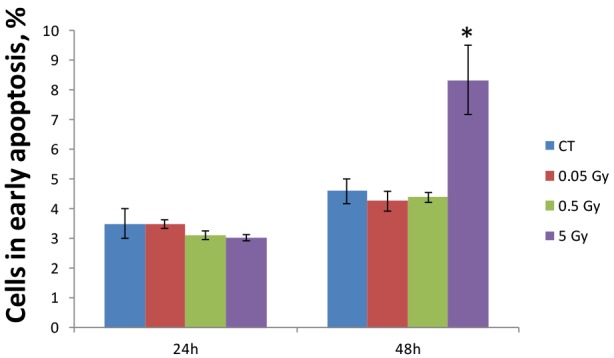
Radiation-induced apoptosis in MCF-7 breast adenocarcinoma cells The number of cells in early apoptosis was measured using the Annexin V-FITC assay for control cells (CT) and cells irradiated with 0.05 Gy, 0.5 Gy, and 5 Gy of X-rays, 24 and 48 hours post exposure. The results are presented as mean values ±S.E.M., n=3. * - significantly different from respective control, p<0.05, Student's t-test.

Figure 4 shows that MCF-7 cells began to undergo early apoptosis 48 hours after irradiation with 5 Gy. Low and medium doses did not cause apoptosis levels different from the control level (Figure 4). In contrast, we found a 1.81-fold increase in Annexin V positive cells 48 hours after exposure to the high dose. These data indicate that MCF-7 breast adenocarcinoma cells can withstand low and medium doses of ionizing radiation and only exhibit apoptotic responses to high doses.

## DISCUSSION

Successful treatment of cancer, including breast cancer, is largely dependent on radiation therapy and proper diagnostics. Radiation therapy is widely used in combination with other treatment modalities, such as surgery, chemotherapy, and hormonal therapy, for treatment of initial and advanced cancers[16, 17]. Choosing the appropriate radiation dose for radiotherapy is vital for achieving the optimal result. Each type of cancer has a different radiosensitivity[18]. Breast cancers are ranked from moderately radiosensitive to radioresistant, therefore requiring higher doses of radiation (45–60 Gy) to achieve a radical cure than many other tumor types. The total dose is divided into 1.8–2 Gy fractions per day for several weeks[19]. There is no data on the effect of low and medium diagnostic doses that might potentially contribute to the severity of malignancy. Overall, data on the effect of different doses of ionizing radiation on tumor cells is scarce.

The purpose of this study was to investigate the radiation-induced gene expression changes in the MCF-7 breast adenocarcinoma cell line. Using microarray technology tools, we were able to screen the differential gene expressions between various radiation doses applied to MCF-7 cells. Here, we report substantial alteration in the expression level of genes after high-dose treatment. In contrast, no dramatic gene expression alterations were noticed after low and medium doses of radiation application. We believe that the ability of the cancer cells to retain their gene expression potential at a constant level, after applying low and medium doses of DNA-damaging radiation insults, means that these doses of ionizing radiation neither contribute to further genomic instability that might result in more severe malignancies, nor cause cell death. Gene expression profiling showed that the expression level of more than 700 genes was changed in the MCF-7 cell line due to 5 Gy X-rays (Figure 1). MCF-7 cells exhibited the expected downregulation of biological pathways, such as cell cycle, DNA replication, DNA repair, and the activation of the p53 pathway (Table 1). Twenty-nine cell cycle regulators where downregulated, which led to cell cycle shutdown. These genes were encoded for cyclins (A2, B1, B2), cyclin-dependant kinases (CDK2, CDK4), cell division cycle proteins (CDC20, CDC25A, CDC7), E2F transcription factors (E2F2, E2F4), mitotic polo-like kinase PLK1, checkpoint kinase CHEK1, mini-chromosome maintenance complex components (MCM 2,3,4,5,6,7), and other cell cycle-associated proteins.

The upregulation of transforming growth factor beta (*TGFB*), and growth arrest and DNA damage-inducible factors (*GADD45A* and *GADD45G*) also contributed to cell cycle deactivation. Obviously, cell cycle deactivation paralleled inhibited DNA replication. Twenty-one genes involved in replication were downregulated: DNA polymerases (A1, A2, D1, D2, E, E2, E3 (except for D4, which was upregulated)), replication factors (*RFC2,3,4,5),* replication protein (*RPA2*), mini-chromosome maintenance complex components (*MCM 2,3,4,5,6,7*), ligase 1, endonuclease *FEN*, and ribonuclease H2 (*RNASEH2A*) (Figure 2).

A specialized DNA damage response was initiated through the activation of the p53 pathway due to the overexpression of BCL2-associated X protein (*BAX*), damage-specific DNA-binding protein *(DDB2*), sestrin1 (*SESN1*), and growth arrest and DNA damage-inducible factors (*GADD45A* and *GADD45G*). DNA repair pathways were downregulated primarily due to the decrease in the expression of specific repair polymerases and replication factors. For instance, base excision repair downregulation was caused by a low expression of polymerases (D1, D2, E, E2, E3), uracil-DNA glycosylase (*UNG*), ligase 1 (*LIG1*), and endonuclease (*FEN1*); NER downregulation was due to the same polymerases and ligase 1, as well as replication factors (*RFC2,3,4,5*) and *RPA2*; MMR pathway deregulation was caused by a low level of *MSH6*, polymerases D1 and D2, *LIG1, RPA2, RFC2,3,4,5,* and exonuclease 1 (*EXO1*); and downregulation homologous recombination pathway was caused by low expression levels of *RAD54L, XRCC3*, polymerases D1 and D2, *RPA2*, Bloom syndrome, RecQ helicase-like (*BLM)*, and topoisomerase (*TOP3A*). Hence it would be important to determine whether severely disrupted pathway activity was correlated with activity and effectiveness of DNA repair in the drug-resistant cell lines.

Gene expression profiling data were confirmed through the qRT-PCR analysis of six genes that were changed into MCF-7 cells after radiation treatment. Polymerases A, D, and E were involved in most of the biological processes that were affected in MCF-7 cells after radiation exposure (Figure 2, Figure 3). As *GADD45G*, Cyclin A, and Aurora B are involved in DNA damage responses, the cell cycle, and cell division, their expression levels were of great interest to us.

Members of the aurora kinases family have been actively studied as mitotic progression targets in cancer. Mutations associated with the aurora gene amplification were reported in human cancers[20]. Tumor development and progression due to aberrant chromosomal segregation and aneuploidy is a common outcome of the misregulation of the Aurora B function[21].

Inhibition of Aurora B during the fractionated radiation treatment suppressed the repopulation of human cancer cells[22]. Similarly, 5-Gy X-rays caused a significant downregulation of Aurora B in drug-sensitive cell lines, which correlated with slower mitotic progression and the suppressed repopulation of the cells. Cyclin A expression was also decreased, which may be associated with a lower DNA replication status and a suppressed cell cycle progression. In addition*, GADD45G*, which is a member of growth arrest and DNA-damage inducible genes, was over-expressed after both 0.5 and 5 Gy of irradiation (Figure 3). This indicates the existence of radiation stress in the cells, which can result in cell cycle arrest, senescence, and apoptosis[23].

Significant downregulation of polymerases A, D, and E confirms the suppression of DNA replication and DNA repair processes. Overall, gene expression profiling and qRT-PCR analysis showed a strong response in MCF-7 cells to high doses of ionizing radiation, allowing us to conclude that these cells were high-dose radiosensitive. In contrast, cells did not respond to low and medium doses of X-rays on the gene expression level, which signifies that they are low-dose radioresistant.

A similar dose-dependent response was seen on the epigenetic level that was tested by the microRNA expression analysis. Radiation-induced changes in miRNA expression usually lead to changes in the synthesis of proteins involved in the main cellular biological pathways. As shown in Table 2, the validated targets of misregulated miRNAs fall in the cell cycle and apoptosis categories (Table 2). For instance, downregulation of miR-106a may inhibit cell proliferation by activation of RB1 tumor suppressor. RB1 is a transcriptional repressor of E2F1 and, when active, leads to cell cycle arrest. Activated transcription of RB1, together with p21 and p16, was shown to suppress tumor cell growth[24]. Another study has reported that an inactive RB1 pathway, a hallmark of cancer, is associated with accumulation of Akt oncogene[25]. As we can see from Table 2, Akt is a validated target of the miR-149, which was upregulated after 5 Gy of X-rays. Akt kinase regulates multiple biological processes such as proliferation, cell survival, growth, and angiogenesis; therefore, its potential inactivation by the epigenetic miRNA mechanism might lead to cell death after high-dose radiation treatment. Similarly, p21, a cyclin-dependent kinase inhibitor, is a target of miR-17 and miR-106b (Table 2). P21 blocks cell cycle progression in response to DNA damage and was shown to be activated after radiation exposure[26]. Another target of miR-17 is pro-apoptotic factor BIM of the Bcl-2 family. BIM induces anoikis through a caspase-mediated pathway and is known to be activated after ionizing radiation exposure[27]. Overall, miRNA analysis has shown dose-dependent radiation-induced miR expression alterations that are associated with cell cycle arrest and cell death. An increased rate of apoptosis was determined by Annexin V assay (Figure 4). Only high dose (5 Gy) radiation led to early apoptosis, 48 hours after radiation treatment.

The results of this study show that high doses of radiation affect gene expression genetically and epigenetically, leading to alterations in cell cycle, DNA replication, and apoptosis. Further investigation is required to reveal the exact molecular mechanisms of such alterations, which would enable the improvement of cancer treatment methods and radiosensitivity.

## MATERIALS AND METHODS

### Cell line and cell culture conditions

The MCF-7 human breast adenocarcinoma cell line was previously developed and described elsewhere [28, 29]. Cells were grown and maintained in Dulbecco's Modified Eagle's Medium (DMEM /F-12) with 2.5 mM L-Glutamine, without HEPES and Phenol Red (HyClone, Logan, UT), supplemented with 10% heat-inactivated fetal bovine serum (HyClone, Logan, UT), in the presence of antibiotics 100 U/mL penicillin and 100 μg/mL streptomycin (Sigma-Aldrich Chemical Co., St. Louis, MO), and in a 5% CO2 atmosphere at 37°C. Cells were harvested for analyses by trypsinization [28, 29].

### Irradiation conditions

Cells were irradiated at 60% confluency in Dulbecco's Modified Eagle's Medium (DMEM). Three radiation doses (0.05, 0.5 Gy and 5 Gy, 90 kV, 5 mA) were applied to check the cellular radiation responses. Unirradiated cells served as the control. Cells were harvested 24 hours and 48 hours after irradiation. All the cells were tested in triplicate. The experiments were independently reproduced twice.

### Whole-genome gene expression profiling

### RNA isolation

Total RNA was isolated using the Illustra RNAspin Mini kit (GE Healthcare Life Sciences, Buckinghamshire, UK). Approximately 5 × 10^6^ cultured cells were processed following the manufacturer's instructions. Samples were eluted in Ultrapure DNase/RNase-free distilled water, which was provided in the kit. RNA samples were quantified using ultraviolet spectroscopy (NanoDrop, Wilmington, DE) and were further assessed for RNA integrity (RIN) on the Aglient 2100 Bioanalyzer (Santa Clara, CA) using the RNA Nano-chip Kit. RNA samples with RIN values of seven or better were used for further analysis.

### Library preparation

CRNA was created using the Ambion Illumina TotalPrep RNA Amplification Kit (Applied Biosystems, Carlsbad, CA) with an input of 500 ng of total RNA per sample. Briefly, oligo-dT primers were used to synthesize first strand cDNA containing a phage T7 promoter sequence. Single-stranded cDNA was converted into a double-stranded DNA template via DNA polymerase. RNase H simultaneously acted to degrade the RNA. Samples of cDNA were purified in filter cartridges to remove excess RNA, primers, enzymes, and salts. The recovered cDNA was subjected to *in vitro* transcription using biotinylated UTPs. This step created, labeled, and amplified cRNA. A final purification step removed unincorporated NTPs, salts, inorganic phosphates, and enzymes, which prepared the samples for hybridization.

### Hybridization and detection

Illumina's direct hybridization assay kit was used to process samples according to the manufacturer's protocol (Illumina, San Diego, CA). Overnight, 750 ng from each cRNA sample was hybridized into the Illumina HumanHT-12_v4 Whole Genome Expression BeadChip arrays. Afterward, a 10-minute incubation with a supplied wash buffer at 55°C preceded a 5-minute room-temperature wash. The arrays were incubated in 100% ethanol for 10 minutes. A second room-temperature wash lasted two minutes with gentle shaking, which completed this high stringency wash step. The arrays were blocked with a buffer for 10 minutes and washed before a 10-minute steptavidin-Cy3 (1:1000) probing. After a five-minute wash at room temperature, the BeadChips were dried and imaged. Six controls were also built into the Whole-Genome Gene Expression Direct Hybridization Assay system to cover aspects of the array experiments, including controls for the biological specimen (14 probes for housekeeping controls), three controls for hybridization (six probes for Cy3-labeled hybridization, four probes for low stringency hybridization, and one probe for high stringency hybridization), signal generation (two probes for biotin control), and approximately 800 probes for negative controls on an eight-sample BeadChip. The arrays were scanned on the iScan platform (Illumina), and data were normalized and scrutinized using Illumina BeadStudio Software.

### BeadChip statistical analysis and data processing

The false discovery rate (FDR) was controlled using the Benjamini-Hochberg method. The Illumina Custom Model took the FDR into account and was used to analyze the data. Differential gene expression (at least a 0.5-fold change) from sham-treated animals was determined to be statistically significant if the *p* value after the Benjamini-Hochberg method adjustment was lower than 0.05. The values were transformed to show a log2 scale.

Lists of regulated transcripts were inserted into the web-based DAVID Bioinformatics Resources 6.7 (NIAID/NIH) Functional Annotation Tool [30, 31]. This program was used to group genes into functionally relevant categories: metabolic processes, transport, response to stimulus/stress, immune response, apoptosis, and cell cycle processes.

### Quantitative real-time PCR

Quantitative real-time PCR was performed to confirm the Whole-Genome Gene Expression results for the regulation direction (either up or down) of select genes. Six genes (aurora B, cyclin A, *GADD45G*, polymerases A, D, and E) were selected from the gene list of significantly differentially expressed transcripts, representing a preliminary review of the acquired gene expression data. 18SrRNA was used as a reference gene. All the reactions were performed using cDNA synthesized from the same RNA extraction as the BeadChip experiments, and 500 ng of the sample was used for the Bio-Rad iScript Select cDNA Synthesis Kit (Bio-Rad Laboratories, Hercules, CA). Samples were stored at −20°C for long-term storage and at 4°C until they were used for subsequent qRT-PCR reactions.

Primers were designed using the NCBI database and PrimerQuest (Integrated DNA Technologies, Inc, Coralville, IA). The following primers were designed: *hAURKB* forward primer (5′-TGA GGA GGA AGA CAA TGT GTG GCA-3′) and reverse primer (5′-AGG TCT CGT TGT GTG ATG CAC TCT-3′); 18SrRNA reference gene primers (5′-GTC AAG TTC GAC CGT CTT CT-3′ and 5′-AGC TTG CGT TGA TTA AGT CC-3′); *CCNA2* forward primer (5′-ATG AGC ATG TCA CCG TTC CTC CTT-3′) and reverse primer (5′-TCA GCT GGC TTC TTC TGA GCT TCT-3′); *hGADD45G* forward primer (5′-TGC TGC GAG AAC GAC ATC GAC ATA-3′) and reverse primer (5′-TCG AAA TGA GGA TGC AGT GCA GGT-3′); *hPOLA1* forward primer (5′-GGC AAT GGC TTT GAA ACC AGA CCT-3′) and reverse primer (5′-ATG CTG AAA GCC ATC ACG ACA AGC-3′); *hPOLD1* forward primer (5′-AAC CTG TGT TAC ACC ACG CTC CTT-3′) and reverse primer (5′-TCC GCA CTG AGG TCT TCA CAA ACT-3′); *hPOLE* forward primer (5′-AGA TTG TGC AGA TCA GCG AGA CCA-3′) and reverse primer (5′-TTA CCT TGC GAT ACG AAG CAC CCT-3′). Reactions were prepared using 1 μL of diluted cDNA, 10 pmol/μL of each forward and reverse primer, and Ssofast EvaGreen Supermix (Bio-Rad Laboratories, Hercules, CA), prepared according to the manufacturer's instructions. Samples were prepared in triplicate and were run on the Bio-Rad C1000 Thermal Cycler equipped with the CFX96 Real-Time System. The qRT-PCR protocol consisted of denaturation at 95°C for two minutes; 43 cycles of denaturation (95C, 5 seconds) and annealing/extension (55°C, 5 seconds); and a final extension at 65°C for five seconds. For every set of primers, annealing temperature optimization, melting curve analysis, and a gel analysis of the amplicon were performed. To evaluate PCR efficiency, a standard curve was established using a series of cDNA dilutions. Data was captured and organized using Bio-Rad CFX Manager 2.1 software (Bio-Rad Laboratories, Hercules, CA).

### QRT-PCR statistical analysis

Quantification data from the Bio-Rad CFX Manager software was analyzed using the Pfaffl method in Microsoft Excel [32]. Graphs showing a fold change from the sham group were created, and transcript regulation directions (up- or downregulation) were matched to the Whole-Genome Gene Expression results.

### miRNA microarray expression analysis

Total RNA was isolated using Trizol reagent (Invitrogen, Burlington, ON) according to the manufacturer's instructions. Approximately 5 × 10^6^ cultured cells were processed following the manufacturer's instructions. One ug of total extracted RNA was sent to LC Sciences (Austin, TX) for miRNA microarray analysis.

### Annexin V assay

For the early detection of apoptosis, an Annexin V-FITC Apoptosis Detection Kit I (BD Biosciences, San Jose, CA) was used according to the manufacturer's protocol. Cells were grown and irradiated as previously described (Section 2.2). The analysis was performed 24 and 48 hours after radiation exposure. Cells were harvested, washed with PBS, resuspended in a 1X binding buffer, stained with Annexin V and propidium iodide for 15 minutes at 25°C in the dark, and analyzed using flow cytometry within one hour at the Flow Cytometry Core Facility (University of Calgary, Calgary, AB). The results were represented as a percentage of gated Annexin V positive cells.
